# The mediating effect of problem-focused coping on the relationship between emotional clarity and mental health among older adults

**DOI:** 10.3389/fnbeh.2024.1465254

**Published:** 2024-10-15

**Authors:** Myung Hyun Cho, Kee-Hong Choi

**Affiliations:** ^1^BK21 FOUR R&E Center for Psychology, Korea University, Seoul, Republic of Korea; ^2^School of Psychology, Korea University, Seoul, Republic of Korea

**Keywords:** emotional clarity, problem-focused coping, life satisfaction, depression, older adults

## Abstract

**Objective:**

Individuals who can recognize emotions well are better able to identify and accept their feelings and manage them. This study examined the mediation of problem-focused coping in the pathway through which emotional clarity predicts higher life satisfaction and lower depression in older adults.

**Methods:**

In total, 150 older adults (75 male and 75 female, aged 60–69 years, with a mean of 64.53 [SD = 2.49]) participated in a face-to-face survey, answering questions on emotional clarity, problem-focused coping, life satisfaction, and depression.

**Results:**

Emotional clarity was associated with higher life satisfaction and lower depression in older adults. People who were aware of their emotions well were in better emotional condition. Mediation analysis revealed that problem-focused coping mediated the positive relationship between emotional clarity and life satisfaction and the negative relationship between emotional clarity and depression. Older adults who understand their own emotions tend to deal with emotional events in a problem-focused manner, leading to high life satisfaction and low depression.

**Conclusion:**

This study identifies cognitive conditions for increasing life satisfaction and preventing depression in later life and offers suggestions for personal and social efforts to maintain mental health.

## 1 Introduction

Recognizing one’s emotions is a fundamental part of the emotional experience and the most important factor in emotional processing (Mayer and Stevens, 1994). By clearly recognizing emotions, individuals are better able to identify and accept their feelings (Kim and Chung, 2016) and manage them during emotional problems (Greenberg, 2004). People who are aware of what they feel are better able to manage their emotions (McFarland and Buehler, 1997). Emotional clarity refers to the clear identification and understanding of emotions in the process of emotion recognition (Boden and Thompson, 2017; Bodena and Berenbaum, 2011; Salovey et al., 1995) and plays an important role in improving mental health and quality of life. Especially in older age, the cognitive functioning of emotions and the recognition and regulation of emotions tend to become more difficult owing to aging effects (Braver and West, 2011; Isaacowitz and Livingstone, 2014; Santorelli and Ready, 2015). Therefore, examining why emotional clarity is important in old age and identifying its mechanisms are important steps in protecting mental health and improving the quality of life in old age. Although the functions of emotion clarity are diverse, this study focused on the basic process by which clear emotion recognition makes individuals objective and cognitive, suggesting that problem-focused coping according to emotion clarity contributes to individual mental health.

### 1.1 Emotional clarity and mental health

Emotional clarity helps one manage, accept, and resolve emotions (Greenberg, 2004; Kim and Chung, 2016; McFarland and Buehler, 1997) and adaptively respond to negative events (Cho and Na, 2017). Emotional clarity predicts mental health. Studies of college students show that those with high emotional clarity felt the academic environment was less threatening and experienced lower psychophysiological stress (Mowrer, 2007). The subtle information provided by paying attention to and understanding one’s emotions facilitates selecting and using emotion regulation strategies (Barrett and Gross, 2001). In older adults, emotional intelligence, including emotional clarity, increases psychological wellbeing by enhancing life satisfaction (Delhom et al., 2017). Older adults who are more likely to pay attention to, understand, and improve their emotions are more likely to lead satisfactory lives and have higher levels of psychological wellbeing. Rey et al. (2011) confirmed the relationship between emotional intelligence and life satisfaction among adolescents, finding that higher levels of mood clarity and emotional repair correlated with higher levels of life satisfaction, mediated by high self-esteem. Wang et al. (2019) found that the relationship between emotional clarity and job satisfaction is mediated by cognitive reappraisal. Emotional clarity contributes to workers’ job satisfaction as they can reappraise problems and experience more satisfying work.

Low emotional clarity lowers psychological wellbeing (Augusto-Landa et al., 2010; Augusto-Landa et al., 2011; Montes-Berges and Augusto-Landa, 2014) and increases depression (Kennedy et al., 2010; Salovey et al., 1995). Kennedy et al. (2010) demonstrated that mood clarity reduces depressive symptoms by mitigating the negative effects of stress, alleviating depressive symptoms by reducing the physical pain associated with stress. Without clear recognition of feelings, one is more likely to be emotionally vulnerable owing to physical discomfort. Guil et al. (2022) found that emotional clarity interacts with emotional attention and emotional repair as emotional intelligence factors, with higher emotional clarity associated with higher emotional attention, promoting emotional repair and reducing depression likelihood. Thompson et al. (2015) showed that the effects of emotional clarity differ according to valence. People with major depressive disorder had particularly low clarity regarding negative emotions, indicating that an inability to recognize one’s inner thoughts during negative emotions can lead to depression.

### 1.2 Emotional clarity and problem-focused coping

Emotional clarity is an objective state that allows us to approach emotional events in a problem-focused rather than an emotional manner. Conceptually, problem-focused coping involves facing a problem and actively trying to solve it (Lazarus and Folkman, 1984) with the goal of either solving the problem itself or replacing perceived stress with something else. Instead of being overwhelmed by emotions, people take an objective view and consider the problem and the best way to solve it. People who are more aware of their emotions tend to exhibit better emotion regulation (Grewal et al., 2006; McFarland and Buehler, 1997). Individuals good at identifying their emotions are more likely to respond actively and plan responses to emotional events, reconstructing and reappraising them positively (Gohm and Clore, 2002; Salovey et al., 1995; Wang et al., 2019). People with high emotional clarity focus on the event triggering the emotion and deal with it actively rather than being overwhelmed by emotions. Delhom et al. (2018) found that emotional intelligence, including emotional clarity, reduces depression through problem-focused coping, confirming that problem-focused coping is essential in emotion regulation. Better understanding of our feelings allows us to identify the problem’s root. For example, recognizing discomfort as anger helps identify its source and use approaches like direct communication or reinterpretation to alleviate the anger. Clear recognition of emotions can lead to problem-focused coping as a mechanism for cognitive emotion regulation.

Emotional clarity is positively related to problem-focused coping. Research shows that people with higher mindfulness levels are more likely to use problem-focused coping strategies (Nevill and Havercamp, 2019; Weinstein et al., 2009). College students who become more aware of their inner thoughts through mindfulness training demonstrate increased problem-focused coping (Halland et al., 2015). Dispositional mindfulness mediates the relationship between problem-focused coping and increased positive affect (Tomlin et al., 2021). People with higher emotional intelligence, including emotional appraisal, engage in problem-focused or rational (task-focused) coping (Delhom et al., 2018; Noorbakhsh et al., 2010; Saklofske et al., 2007). Alexithymia research shows that low alexithymia predicts higher task-oriented or problem-focused coping (Besharat, 2010; Parker et al., 1998; Taskin Yilmaz et al., 2023; Vingerhoets et al., 1995). Emotional clarity contributes to mental health through problem-focused coping strategies.

### 1.3 Problem-focused coping and mental health

Problem-focused coping predicts mental health, life satisfaction, and protection against depression. Reyes et al. (2021) found that older adults using problem-focused coping had higher subjective wellbeing, while those using emotion-focused coping had lower subjective wellbeing. Chen et al. (2018) showed that problem-focused coping mediated the relationship between age and positive affect. Although problem-focused coping positively predicted positive emotions, the tendency to engage in problem-focused coping decreased with age. This highlights the need to promote problem-focused coping to increase positive emotions later in life.

Problem-focused coping mitigates emotional distress. Chen and Sun (2019) found that problem-focused coping prevented cumulative risks, including family conflicts, health problems, and work stress, from leading to depression. Cong et al. (2021) found that problem-focused coping decreases depression among adolescents, with higher levels of problem-focused coping correlating with higher self-esteem and fewer depressive symptoms. High emotion-focused and low problem-focused coping predict depression in adolescents (Breton et al., 2015; Undheim et al., 2016).

Cognitive emotion regulation, focusing on cognitive processes rather than emotions, is protective against depression. Cognitive emotion regulation, such as acceptance, rumination, and positive reappraisal, is associated with fewer depressive symptoms (Garnefski and Kraaij, 2006). Meaning-Centered Coping reduces stress, anxiety, and depression in the context of COVID-19 (Eisenbeck et al., 2021). These findings suggest that problem-focused coping increases psychological wellbeing in a proactive and planned manner (Folkman et al., 1987) and support the finding that cognitive functioning is strongly associated with life satisfaction and positive emotions (Jones et al., 2003). Problem avoidance increases the extent to which wellbeing is reduced during stress (Chao, 2011), and problem-focused coping minimizes this catastrophic outcome.

### 1.4 The present study

This study tested the hypothesis that emotional clarity in later life acts as a protective factor for mental health by increasing life satisfaction and reducing depression through problem-focused coping. Based on research showing that emotional intelligence, including emotional clarity, is related to problem-focused coping, we examined the mediating effects of problem-focused coping. We also tested whether problem-focused coping mediated these relationships, ultimately contributing to maintaining a satisfactory life and preventing depression.

*H1*: Emotional clarity is positively related to life satisfaction.

*H2*: Emotional clarity is negatively related to depression.

*H3*: Problem-focused coping mediates the positive relationship between emotional clarity and life satisfaction.

*H4*: Problem-focused coping mediates the negative relationship between emotional clarity and depression.

We examined mental health in old age based on life satisfaction and depression. Many studies have used high life satisfaction and low depression levels as indicators of mental health (Guney et al., 2010; Headey et al., 1993; Renshaw and Cohen, 2014). Although these factors are independent concepts, life satisfaction is often negatively related to depression (Guney, 2009), which inevitably leads to pathological symptoms (Horstmanshof et al., 2008). Dealing with both factors is necessary to observe and explain mental health. Unlike the past focus on negative mood-based psychopathology to explain mental health, the two-factor view of highly positive mood with low psychopathology is becoming more convincing (Suldo and Shaffer, 2008). Therefore, this study examined mental health using life satisfaction and depression as representative variables of positive and negative states.

## 2 Methods

### 2.1 Participants

In total, 150 older adults (75 male and 75 female) in their 60s in South Korea completed a survey packet through a research company. Their ages ranged from 60 to 69 years, with a mean of 64.53 (SD = 2.49). The age of old age was limited to the 60s in this study because it was judged that people would strongly perceive later life as the age of 60, as that is generally the starting point of retirement and entering old age. The survey was conducted as a face-to-face survey in which participants visited the research company in person and answered the questionnaire provided to them while the surveyor explained the survey questions.

### 2.2 Measurements

#### 2.2.1 Emotional clarity

The Trait Meta-Mood Scale, developed by Salovey et al. (1995) and translated into Korean by Lee and Lee (1997), was used. This scale comprises 13 items on emotional attention, 11 items on emotional clarity, and 6 items on emotional repair, totaling 30 items on a 5-point scale that measures the degree of emotional intelligence through the degree of individual attention, clarity, and belief in improvement related to emotional cognition. This study used only 11 items of the Emotional Clarity scale (e.g., “I almost always know exactly how I feel” and “Sometimes I do not know how I feel” [reverse scored]). *α* = 0.666.

#### 2.2.2 Problem-focused coping

The Coping Scale, developed by Staudinger and Fleeson (1996) and translated into Korean by Ryu (2005), was used to assess problem-focused coping. This scale was originally developed with 13 items, each representing an independent factor (comparison with the past, keep going, distraction, adaptation to the given, faith, wish for information, social support, ups and downs, giving up, life loses meaning, comparison with others, someone else to take over, and humor). Ryu (2005) added one additional item for active coping, and the 14 factors were re-categorized into four semantic domains through factor analysis: problem-focused coping (four items), self-defensive coping (four items), cognitive coping (two items), and passive-dependent coping (four items). This scale is sophisticated in that it proposes more diverse and specific forms of coping, rather than the dichotomy of problem-and emotion-oriented coping in general. To determine the level of problem-focused coping in this study, four problem-focused coping items and two cognitive coping items were extracted from the 14 items (thus, we used six items). Although Ryu (2005) separated problem-focused and cognitive coping as distinct factors, both address strategies to manage the problem itself. For example, item 3 (problem-focused coping) states, “I find that I can better face up to dealing with a difficult situation if I remind myself that I have already solved many other problems in my life,” while item 7 (cognitive coping) states, “I try to find out about all sides of a problem.” Moreover, the “problem-centered coping” that this study aims to confirm is centered on an active approach to confronting and solving problems rather than avoiding them (Lazarus and Folkman, 1984). Given the conceptual overlap in this context, this study judged that combining four items about problem-focused coping and two items about cognitive coping would be more reliable in confirming problem-focused coping. The scales were all reported on a 5-point scale, and Cronbach’s alphas were 0.746 for original problem-focused coping, 0.711 for cognitive coping, and 0.819 for combined problem-focused coping. The factor analysis results for the combined scale were as follows: χ^2^(9) = 25.2 (*p* = 0.003), CFI = 0.942, TLI = 0.904, SRMR = 0.0473, and RMSEA = 0.109.^1^ For simplicity, we use the term “problem-focused coping” in this study.

#### 2.2.3 Life satisfaction

This study used the Life Satisfaction Scale developed by Diener et al. (1985) and translated into Korean by Zho and Cha (1998). The scale is a 5-item, 5-point scale with statements such as “I am satisfied with my life” and “My life conditions are very good.” *α* = 0.833.

#### 2.2.4 Depression

The Short Form of Geriatric Depression Scale, developed by Yesavage et al. (1983) and translated into Korean by Cho et al. (1999), was used. The scale comprises items such as “Do you feel that your situation is hopeless?” and “Are you generally cheerful?” (reverse scored) to measure overall depression levels in old age. It is a 15-item, 15-point scale with a yes (1) or no (0) response per item. The scores are summed to determine the level of depression, with higher scores indicating higher levels of depression. α = 0.865.

### 2.3 Analytical overview

The hypotheses were tested using SPSS version 22.0 (IBM, Armonk, NY, United States). Descriptive statistics checked participants’ characteristics, and a correlation analysis checked relationships among the main variables. A hierarchical regression analysis calculated the variance inflation factor and tolerance to identify whether multicollinearity existed among the predictors of the dependent variables. Kurtosis and skewness were assessed to verify the normality of the data. Finally, the mediating effect of problem-focused coping on the effect of emotional clarity on life satisfaction and depression was tested using Hayes (2013) PROCESS macro model 4. The significance of the indirect effect was determined by checking whether the upper and lower boundaries contained zero by bootstrapping to 5,000 and setting the confidence interval to 95%.

### 2.4 Ethical considerations

All participants provided informed consent, and this study was conducted in accordance with the appropriate ethical regulations of Sogang University, South Korea. Since this study received official academic support, following a review by the National Research Foundation of Korea, the separate IRB approval process from Sogang University was waived.

## 3 Results

### 3.1 Relationships among main variables

Table 1 presents the correlations and descriptive statistics for the variables. There was a significant positive correlation between emotional clarity and life satisfaction (*r* = 0.33, *p* < 0.01) and a significant negative correlation between emotional clarity and depression (*r* = −0.47, *p* < 0.01) among older adults, supporting H1 and H2. Emotional clarity was positively related to problem-focused coping (*r* = 0.49, *p* < 0.01), suggesting that people more aware of their emotions tend to adopt a more problem-focused approach to coping with problems. Problem-focused coping was positively related to life satisfaction (*r* = 0.34, *p* < 0.01) and negatively related to depression (*r* = −0.50, *p* < 0.01), suggesting that older adults who adopt a problem-focused approach to stressful events are more satisfied with their lives and experience less depression. Hierarchical regression analysis tested for multicollinearity among the main predictors. When life satisfaction and depression were entered as dependent variables, all variance inflation factors were below 10 (1.32 and 1.32, respectively) and all tolerances were above 0.01 (0.76, respectively), confirming no multicollinearity problem between emotional clarity and problem-oriented coping on life satisfaction and depression.

**Table 1:** Correlation analyses.

	1	2	3	4	5	6
1. Emotional clarity	1					
2. Problem-focused coping	0.49^**^	1				
3. Life satisfaction	0.33^**^	0.34^**^	1			
4. Depression	−0.47^**^	−0.50^**^	−0.54^**^	1		
5. Gender	0.08	0.1	0.17^*^	0.05	1	
6. Age	−0.01	−0.06	−0.01	0	−0.27^**^	1
Mean	3.46	3.83	4.29	3.62	1.5	64.53
SD	0.44	0.61	1.21	3.69	0.5	2.49
Skewness	0.32	−0.44	−0.22	1.1	0	0.07
Kurtosis	1.07	1.17	0.06	0.36	−2.03	−0.93

^*^p < 0.05, ^**^p < 0.01.

### 3.2 Mediating effects of problem-focused coping

#### 3.2.1 Mediating effect on the relationship between emotional clarity and life satisfaction

The mediating effect of problem-focused coping on the positive relationship between emotional clarity and life satisfaction was confirmed (B = 0.32, Boot SE = 0.15, 95% CI [0.07, 0.65]), supporting H3. Emotional clarity significantly predicted problem-focused coping (B = 0.67, *p* < 0.001) and problem-focused coping significantly predicted life satisfaction (B = 0.46, *p* < 0.01). When controlling for problem-focused coping, the direct effect of emotional clarity on life satisfaction prediction was significant (B = 0.59, *p* < 0.05). The total effect of emotional clarity on life satisfaction was also significant (B = 0.90, *p* < 0.001) (Table 2).

**Table 2:** Mediating effect on life satisfaction.

	*B*	*SE*	*t*	95% CI	
				Lower	Upper
Emotional clarity → Problem-focused coping	0.67	0.10	6.89^***^	0.49	0.88
Problem-focused coping → Life satisfaction	0.46	0.17	2.66^**^	0.12	0.80
(Direct) Emotional clarity → Life satisfaction	0.59	0.24	2.43^*^	0.11	1.06
(Total) Emotional clarity → Life satisfaction	0.90	0.21	4.22^***^	0.48	1.32

*p < 0.05, ^**^p < 0.01, ^***^p < 0.001.

#### 3.2.2 Mediating effect on the relationship between emotional clarity and depression in older adults

**Figure 1:**
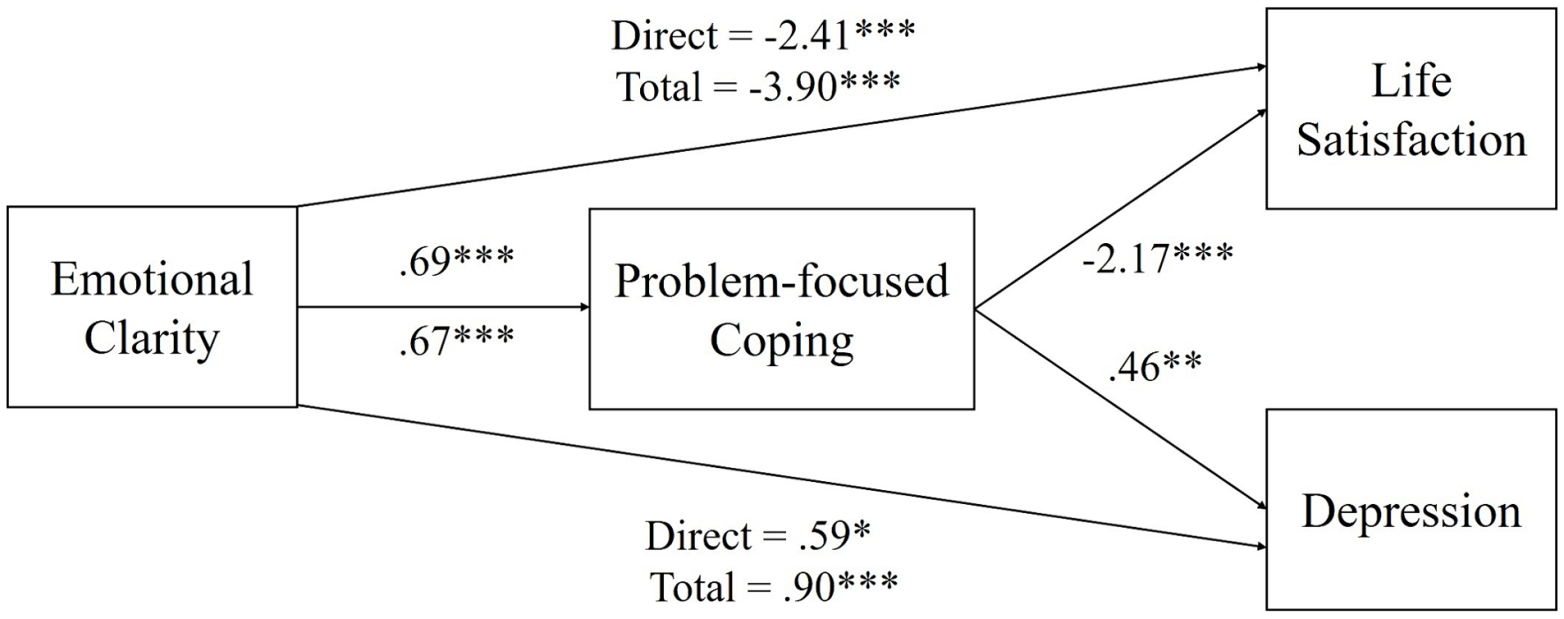
The mediation model of this study. ^*^p < 0.05, ^**^p < 0.01, ^***^p < 0.001.

Consistent with H4, the mediating effect of problem-oriented coping on the negative relationship between emotional clarity and depression was confirmed (B = −1.49, Boot SE = 0.34, 95% CI [−2.15, −0.82]). Emotional clarity predicted problem-focused coping (B = 0.69, *p* < 0.001), and problem-focused coping significantly predicted depression (B = −2.17, *p* < 0.001). When controlling for problem-focused coping, the direct effect of emotional clarity on depression was significant (B = −2.41, *p* < 0.001). The total effect of emotional clarity on depression was also significant (B = −3.90, *p* < 0.001) (Table 3; Figure 1).

**Table 3:** Mediating effect on depression.

	B	SE	*t*	95% CI	
				Lower	Upper
Emotional clarity → Problem-focused coping	0.69	0.10	6.89^***^	0.49	0.88
Problem-focused coping → Depression	−2.17	0.47	−4.60^***^	−3.10	−1.24
(Direct) Emotional clarity → Depression	−2.41	0.66	−3.68^***^	−3.71	−1.12
(Total) Emotional clarity → Depression	−3.90	0.61	−6.41^***^	−5.10	−2.70

^***^p < 0.001.

## 4 Discussion

The main findings are as follows. First, emotional clarity in later life predicts higher life satisfaction and lower levels of depression. Older adults who were better at interpreting and understanding their emotions tended to be more satisfied and had lower levels of depression. These findings support those of research showing that emotional clarity increases life satisfaction and reduces depression in various populations (Delhom et al., 2017; Guil et al., 2022; Günther et al., 2016; Honkalampi et al., 2000; Kennedy et al., 2010; Rey et al., 2011; Saarijärvi et al., 2001; Thompson et al., 2015; Wang et al., 2019) and reaffirm the relationship between emotional clarity and mental health, even in older age when people experience emotional changes. The findings are consistent with existing research suggesting that being aware of one’s internal emotional dynamics helps individuals manage, accept, and resolve emotions (Greenberg, 2004; Kim and Chung, 2016; McFarland and Buehler, 1997).

Second, problem-focused coping mediated the positive relationship between emotional clarity and life satisfaction in later life. Clearly recognizing one’s emotions leads a person to pay more attention to and directly address problems, ultimately leading to life satisfaction and happiness. The results support studies showing the positive effects of problem-focused coping in older adults (Chen et al., 2018; Reyes et al., 2021), suggesting that clear recognition and understanding of one’s emotions are necessary to use a problem-focused coping strategy. Perceiving that one’s overall life is going well requires the ability to adapt to problems needing solutions. Proactively dealing with problems reduces ambiguity, leading to a more stable state and increasing the likelihood of resolution. Recognizing one’s emotional state is necessary for this process. This study suggests that a clear perception of one’s emotions can lead to a more proactive attitude toward problems, resulting in a more fulfilling life.

Third, problem-focused coping mediated the negative relationship between emotional clarity and depression in later life. Depression in later life is not only triggered by aging or illness (Beekman et al., 1995; Leibson et al., 1999) but also by social factors such as loss, loneliness, and low activity (Burnette and Mui, 1996; Cacioppo et al., 2006; Heikkinen and Kauppinen, 2004; Hong et al., 2009). The results suggest that emotional clarity and subsequent problem-focused coping are critical for overcoming emotional vulnerability during personal and environmental changes in later life. Recognizing one’s emotions and dealing directly with problems, rather than avoiding them, may help prevent depression.

### 4.1 Implications

This study offers valuable insights into cognitive and emotional coping that enhance quality of life in later life, emphasizing the role of problem-focused coping and emotional clarity. Clear perceptions of emotions contributed to more positive and fewer negative emotional experiences, reinforcing the beneficial impact of problem-focused coping on mental health. These findings have clinical implications, providing clues for increasing life satisfaction and reducing depression among older adults, thereby promoting a mentally healthy and fulfilling life. Understanding and interpreting emotions are crucial for making informed decisions, particularly in conflict scenarios, and are key to achieving emotionally stable outcomes. This challenge is more pronounced in older adults, in whom cognitive decline can hinder emotion recognition. Emphasizing emotional clarity and problem-focused coping offers a guide for managing emotions in old age and effectively interacting with the environment. This study informs ways to improve older adults’ emotional clarity. For example, community programs including group discussions, emotional literacy workshops, and tailored mindfulness training can be designed. Activities that promote active social participation and emotional interaction with others can help older adults experience a wider range of emotions in their daily lives. At the clinical level, evidence-based programs (e.g., community programs based on cognitive behavioral therapy, emotional monitoring, or autobiographical memory training programs) can be implemented to enhance older adults’ cognitive abilities, thereby enhancing their emotional clarity. These findings may also be applicable beyond older adults, offering direction for clinical and educational interventions for other groups facing emotional challenges, such as individuals with high trait anxiety or children who lack self-regulation skills. Future studies should investigate the universal importance of managing and regulating emotions for a more stable and happier life using diverse samples.

### 4.2 Limitations

This study has some limitations that should be addressed in future research. First, this was a questionnaire-based study, which limits our ability to infer causation between study variables. Factors such as emotional clarity and problem-focused coping require behavioral changes and observations. Questionnaire responses are limited in that they confirm behavioral tendencies rather than actual changes in individuals. Moreover, data were collected at a single time point; therefore, participants’ state of mind during the survey may have affected their responses. Future studies should adopt more empirical methods, such as mindfulness interventions or longitudinal designs, to reach more realistic conclusions based on behavioral observations. Second, this study analyzed older adults in their 60s, but this age group may not be representative of all older adults. Considering the increasing global life expectancy, future studies should include older adults over 70-years-old. Third, when using the problem-focused coping scale, items from the existing scale were re-sorted to create a new scale. While this scale satisfies the model fit and identifies relevant factors, more reliable results could be achieved by utilizing a more structured and validated scale. Future researchers should more precisely conceptualize problem-focused coping and incorporate measures that reflect more realistic behavioral patterns. Fourth, this study focused on the South Korean population, limiting its generalizability to other cultural or geographical populations, including in Western countries. Specifically, alexithymia is higher in Asian countries than in Western countries (Dere et al., 2012; Le et al., 2002). Future studies should include culturally diverse settings. Additionally, since the scale was translated into Korean, it is necessary to examine whether cultural influences affected its validity. Finally, the objective of emotional clarity must be expanded to include other people. Although mental health (life satisfaction and depression) and problem-focused coping tested in this study are internal factors, they are ultimately predicated on interaction with the environment, including interaction with others. Therefore, a clear understanding of not only one’s own emotions but also the emotions of others will contribute to a comprehensive interpretation of situations. Future research could extend the current findings to examine how emotional clarity affects emotion-focused coping strategies. According to socioemotional selectivity theory, older adults focus more on stability and regulation at the emotional level than on acquiring or achieving cognitive information (Carstensen et al., 2003). Therefore, emotion-focused coping also appears to be adaptive in older age, and a comparison with problem-focused coping may provide a more elaborate picture of the impact of coping strategies on mental health.

## 5 Conclusion

In conclusion, it is essential to focus on enhancing emotional clarity later in life. Given the high prevalence of alexithymia among older adults, individual and social efforts are required to support cognitive improvement. This includes creating a supportive environment through community programs, mindfulness training, and active social participation. Both social education and institutional support are crucial in aiding older adults to become more aware of their feelings and thoughts, thereby enhancing their quality of life.

## Data Availability

The datasets presented in this study can be found in online repositories. The names of the repository/repositories and accession number(s) can be found at: https://osf.io/xhdu9/.

## Footnotes

^1^The RMSEA value is somewhat high, which may be attributed to the small sample size (N = 150). When the sample size is limited, model fit should not be evaluated with the RMSEA value (Kenny et al., 2015). However, the SRMR index was 0.0473, which satisfies the goodness-of-fit criteria of being less than 0.05 (Byrne, 2012) or 0.08 (Hu and Bentler, 1999). The $\frac{\chi^{2}}{df}$ value was 2.8, which satisfies other goodness-of-fit criteria as it does not exceed 5 (Carmines and McIver, 1981; Wheaton et al., 1977). Therefore, the newly combined scale was deemed appropriate.
